# Annual acknowledgement of manuscript reviewers

**DOI:** 10.1186/1471-2121-14-10

**Published:** 2013-04-04

**Authors:** Christopher Morrey

**Affiliations:** 1 BioMed Central, Floor 6, 236 Gray's Inn Road, London WC1X 8HB UK

## Abstract

**Contributing reviewers:**

The editors of BMC Cell Biology would like to thank all of our reviewers who have contributed to the journal in Volume 13 (2012).

## 

Cristina Albanesi

Italy

Mohammed Al-Gayyar

Egypt

Hideo Baba

Japan

Susan Bailey

USA

Susan Baker

USA

Abby Benninghoff

USA

Rebecca Berdeaux

USA

Ramesh Bhonde

India

Guylain Boulay

Canada

Claudio Brancolini

Italy

Tim Brummendorf

Germany

Matthew Buechner

USA

Folma Buss

UK

Sara Cabodi

Italy

Mario Cannas

Italy

Carolyn Anne Carr

UK

Namas Chandra

USA

Zhijie Chang

China

Christos Chatziantoniou

France

Jianquan Chen

USA

Ye-Guang Chen

China

Shu-Jun Chiu

Taiwan

Diana Chu

USA

Pin Ju Chueh

Taiwan

Chang Chung

USA

Lucia Cilenti

USA

Audrey Claing

Canada

Andrea Clocchiatti

Italy

Miriam Cnop

Belgium

Philip Cohen

UK

Christoph Daniel

Gibraltar

Dawn Davies

UK

Maria Angeles De La Torre-Ruiz

Spain

Peter Devreotes

USA

Adeline Divoux

USA

Bernhard Dobberstein

Germany

Zhen Dou

China

Arnaud Echard

France

Rita Falcioni

Italy

Ricardo Figueroa

Sweden

Bertrand Fourcade

France

Umberto Galderisi

Italy

Julie Gavard

France

Zafiroula Georgoussi

Greece

Giuseppina Giglia-Mari

France

Deborah Goberdhan

UK

Margarete Goppelt-Struebe

Germany

Cara Gottardi

USA

Christopher Heinen

USA

Juergen Heinisch

Germany

Janet Henderson

Canada

Alexander Hergovich

Switzerland

Dominique Heymann

France

Olaf Hopfer

Germany

Yujie Huang

USA

Masaki Inagaki

Japan

Michel Jadot

Belgium

Abdul Hamid Jan

Pakistan

Changjiang Jin

China

Nicole Kane

UK

Jack Kaplan

USA

Marianna Karagianni

Germany

Gregory Kitten

Brazil

Etsuko Kiyokawa

Japan

Jorg Kleeff

Germany

Aikaterini Kontrogianni-Konstantopoulos

USA

Pavel Kovarik

Austria

Gerd Krause

Germany

Shaun Kunisaki

USA

Joseph C K Leung

Hong Kong

Fiona Lewis

UK

Barbara Lewko

Poland

Guo-Min Li

USA

Dan Lindholm

Finland

Georg Lüers

Germany

Julian Lum

Canada

Nancie Maciver

USA

Tatsuya Maeda

Japan

Adriano Marchese

USA

Lydia Matesic

USA

Karl Matter

UK

Irene Mavelli

Italy

Joachim Meissner

Germany

Andre Menke

Germany

Andreas Merdes

France

Judith Miklossy

Switzerland

Teresa Moloney

Ireland

Mervyn Monteiro

USA

Noel Morgan

UK

Ciaran Morrison

Ireland

Tatsuji Nishihara

Japan

Angelika A Noegel

Germany

Hajime Ohgushi

Japan

Yash Patel

USA

Marco Patruno

Italy

Melissa Petreaca

USA

Didier Pisani

France

Roberta Piva

Italy

Izabela Podgorski

USA

Martin Pool

UK

Randy Poon

Hong Kong

Jyothi Prasanna

India

Claudia Puri

UK

Mrinalini Rao

USA

Krishanu Ray

India

Guillermo Romero

USA

Douglas Ruden

USA

Tarek Samad

USA

Gonzalo Sanchez-Duffhues

Netherlands

Andrew James Sanders

UK

Alireza Sarvestani

USA

Amy Sater

USA

Andrea Sgorbissa

Italy

Adam Sharples

UK

James Sherley

USA

Xiaobing Shi

USA

Andrew Smith

UK

Silvia Soddu

Italy

Ylermi Soini

Finland

Stephen Somkuti

USA

Igor Stagljar

Canada

George Stark

USA

Ursula Stochaj

Canada

Masami Suganuma

Japan

Yong Sun

USA

Anders Sundqvist

Sweden

Alexandra Surcel

USA

Hiroshi Takemori

Japan

Qi-Qun Tang

China

Mohamed Trebak

USA

Sergey Troyanovsky

USA

Hiroyuki Tsuchiya

Japan

Nasir Uddin

Pakistan

Gyorgy Varo

Hungary

Caroline Van Haaften

Netherlands

Christina Van Itallie

USA

Pamela Vandevord

USA

Tibor Vellai

Hungary

Alexey Vereninov

Russian Federation

Maria Vinci

UK

Anne Kathrin Voss

Australia

Limin Wang

USA

Tarsha Ward

USA

Cheryl Waring

UK

Gregory Watson

Australia

Kees Weijer

UK

Fu-Qiang Wen

China

Paul Whitley

UK

Xuechun Xia

China

Jin-Ming Yang

USA

Cristina Zanini

Italy

Zhuo Zhang

USA

Xiaoping Zhong

USA

